# The Control Motivation Function of Populist Attitudes: Causal Evidence that Populist Reasoning Raises Perceptions of Control

**DOI:** 10.1177/01461672251373106

**Published:** 2025-10-31

**Authors:** Annedore Hoppe, Immo Fritsche, Helena Pauen

**Affiliations:** 1 University of Leipzig, Germany

**Keywords:** populism, populist attitudes, populist reasoning, control perception, social identity

## Abstract

The present studies investigate how populist attitudes influence perceptions of personal control. Populist reasoning is proposed to enhance personal control by promoting a sense of belonging to an agentic group (“the people”) that opposes a perceived antagonistic “corrupt elite.” Across three experiments (*N* = 733), participants were asked to adopt either a populist or a pluralistic reasoning style. In Study 3, the salience of high versus low personal control was additionally manipulated. Results showed that populist reasoning increased participants’ sense of control, particularly in relation to specific goals, such as performing well in an upcoming debate. However, making low control salient tended to diminish this effect. These findings suggest that populist narratives may be appealing during crises due to their control-enhancing function. The novel experimental method for manipulating populist attitudes opens new pathways for testing causal effects and understanding the motivational drivers of populism, potentially contributing to future interventions in this field.

What is the psychological function of populist attitudes? Mudde and Kaltwasser (2017) define populist attitudes as the belief that the “pure people” is in a struggle with corrupt elites and that the general will of the people should be directly expressed in politics. Political sciences have described a recent rise of populist political movements shattering Western societies and their political systems (Bernhard & Kriesi, 2019), often attributed to societal crises and peoples’ perception of personal threat (Brubaker, 2017; Dennison & Turnbull-Dugarte, 2022; Lüders et al., 2021). We argue that people turn to populism to experience a sense of control through the self, inherent in populist beliefs of collective agency. Accordingly, we hypothesize populist attitudes to enhance people’s personal control perceptions. Specifically, populist ideology construes a self-inclusive social group (“the people”) that is characterized as a collective actor, destined to pursue its legitimate goals, manifested in the battle against evil (“the corrupt elites”). The current article introduces the novel group-based control (Fritsche, 2022) account of populist attitudes and presents, for the first time, three causal experimental tests of whether populist attitudes increase perceptions of control.

## Populism and Populist Attitudes

Political sciences have defined populism in various ways. Probably, the most widespread, shared, and influential definition comes from Mudde and Kaltwasser (2017, p. 6). Here, populism is conceived of as “... thin-centered ideology that considers society ultimately separated into two homogeneous and antagonistic camps, ‘the pure people’ versus ‘the corrupt elite’ and which argues that politics should be an expression of the volonté générale (general will) of the people.” More recently, Wuttke et al. (2020) summarized definitions of populism as typically representing the three factors anti-elitism, popular sovereignty, and Manichean outlook (dualistic worldview, in which the world is divided into two clearly separate, morally opposed camps, e.g., “good” vs. “evil.”). There might be right-wing or left-wing versions of populist attitudes that differ, for instance, regarding whether the “people” is defined as an ethnic (i.e., the ethnic Germans) or a socioeconomic ingroup (i.e., the ordinary people). At the same time, populist thinking might be related to other ideologies or specific concerns, for instance, preserving the welfare state.

According to Laclau (2005), populism can be considered as a result of unfulfilled demands of the people leading to an emerging gap between “the people” and “the elite.” Formerly heterogeneous subgroups find common ground through their dissatisfaction. The antagonistic entity (“Elite”) is either unwilling or unable to fulfill these demands and is therefore morally devalued. Müeller (2017) goes one step further and argues that populist groups are a threat to modern democracies due to populist groups’ claim to exclusively represent “the people,” neglecting oppositions, and excluding other opinions and interests that are usually valued in pluralist societies.

Populist leaders may encourage a sense of injustice and victimhood even in times of economic prosperity by framing objective relative gratification as relative deprivation (Mols & Jetten, 2016). Populism can be considered as a rhetorical mechanism where political leaders make use of emotionally and morally charged crises narratives. A populist leader is then presented as the supposed solution to the crisis (Homolar & Scholz, 2023; see also Bonansinga, 2022). In a similar manner, Moffitt and Tormey (2014) emphasize that populism can be considered as a political style that includes that populist leaders appeal to “the people” through emotional and crisis rhetoric. Populists also emphasize their closeness to the people through slang, political incorrectness or contempt for technocratic language. Similar to Mudde (2004), it is also assumed here that populism is not linked to a specific political ideology.

In general, opinions differ on whether populism benefits or harms democracies. Possible positive effects are sometimes attributed to left-wing populism, whereas damage is mostly considered in relation to right-wing populism (see Priester, 2007). Mouffe (2000), for instance, considers populism as a form of democratic renewal that promotes political participation and agonistic exchange. Here, populism is seen as an important part of vibrant democracies, as it accepts conflict as part of democracies. In contrast, right-wing populist movements are often discussed as a threat to liberal democracies, given their tendency to neglect fundamental human rights, minority rights, and democratic principles, such as the reconciliation of different interests.

Phenomena, as the “Brexit” or the first U.S. presidency of Donald Trump, raised a public interest in explaining populism. This fueled initial psychological research on its motivational underpinnings, demonstrating that populist attitudes are linked to high social dominance orientation (Forscher & Kteily, 2020), authoritarian aggression (Womick et al., 2019) and to low agreeableness (Bakker et al., 2016). However, so far, psychological research has fallen short in experimentally testing causal effects of populism. Also, populist attitudes have often been operationalized in a fuzzy way, such as attitudes toward a political actor deemed populist. For a first effort to investigate populism as an experimental factor, we relied on the definition given by Mudde and Kaltwasser (2017) as it currently seems to be the most influential definition of populism. Our aim was to test the psychological effects of this kind of populist belief.

Specifically, we inquired whether *adopting* populist attitudes increases personal control perceptions. This hypothesis is derived from previous research suggesting that people are more inclined toward populist ideology when they have to cope with societal crises and personal deprivation (Brubaker, 2017; Priester, 2007; Spruyt et al., 2016) that might shatter their sense of personal control. Specifically, populist attitudes are correlated with the perception of societal crisis (e.g., climate crisis, pandemic, economic crises; Dennison & Turnbull-Dugarte, 2022) and anger about the economic situation (Rico et al., 2017). Furthermore, perceptions of personal relative deprivation (Lüders et al., 2021; Spruyt et al., 2016; Staerklé et al., 2022), social exclusion (Langenkamp & Bienstman, 2022; Manunta et al., 2022; Obradović et al., 2020) and little personal influence on political decisions (Spruyt et al., 2016) were found to be related to populist attitudes. Explanations discussed highlight different psychological needs (Deci & Ryan, 2000; Fiske, 2018). For instance, turning to populism may give threatened people a positive social identity (“We, the good people”) and restores self-esteem (Spruyt et al., 2016). Also, being part of a populist ingroup may satisfy people’s need to belong (Goodman, 2022). Other explanations rely on the reduction of control concerns, such as a regaining of power after being exposed to the narrative of humiliation (Homolar & Löfflmann, 2021), or uncertainty concerns, such as the provision of clear guidelines which help to reduce the overwhelming complexity of societal change. Then, populism may simply promise a return to the “proper” order of the community (Heinisch & Jansesberger, 2024).

We aim to test a control motive explanation of populist responses. We propose that being populist helps people to experience personal control over the environment. Based on a novel group-based control perspective (Fritsche, 2022), we propose that adopting populist attitudes should increase personal control perceptions through a sense of belonging to an agentic group. Thus, the focus of our research is neither populist leaders or parties, nor the populist rhetoric of specific politicians or parties. We suggest that people’s personal populist attitudes (here elicited by populist reasoning in an experimental manipulation) increase their personal perceptions of control.

## Social Identity and Group-Based Control

According to social identity theory (Turner et al., 1987), individuals can either define themselves as an individual or as a member of a social group. This so-called *social identity* is defined as part of people’s self-concept that derives from their knowledge of personal group membership together with the emotional value attached to that membership (Tajfel, 1981).

Perceiving the self as an autonomous agent having an impact on its environment (i.e., as having control) is a basic human need (Deci & Ryan, 2000; Fiske, 2004; Hornsey et al., 2019) and crucial for health and personal performance (Relke et al., 2022; Skinner, 1996). Group membership and group-based action seem to be important providers of subjective control, as proposed by group-based control theory (Fritsche, 2022; Fritsche et al., 2013). People often think of groups as agents and not just as categories (Brewer et al., 2004). Collective agency (i.e., control) means that the group is assumed to have autonomous joint goals, is pursuing its goals, and is likely to affect its environment (Fritsche & Masson, 2021). Thus, identifying as a member of an agentic group can positively affect people’s personal sense of control through the process of self-stereotyping (Hogg & Turner, 1987). Consequently, when people feel deprived of personal control (e.g., under conditions of crisis), they may identify with an agentic ingroup (e.g., their ethnic group) or demonstrate collective agency to regain personal control. In line with that, previous research demonstrated that salient control threat increases people’s identification with agentic ingroups (Proudfoot & Kay, 2018; Stollberg et al., 2015), ingroup bias (Fritsche et al., 2013), as well as thinking and acting in terms of group membership (Lautenbacher et al., 2025; Stollberg et al., 2017, 2024). In turn, salient ingroup identification (Greenaway, 2024; Greenaway et al., 2015) and collective control (Jugert et al., 2016; Relke et al., 2022, 2024) increased people’s sense of personal control.

## Populist Attitudes and Control Perceptions

From a group-based control perspective, populist attitudes may provide people with a sense of personal control via subjective membership in an agentic group. This is because, according to its pertinent definitions (Mudde & Kaltwasser, 2017; Wuttke et al., 2020) populist reasoning contrasts a seemingly highly inclusive and homogeneous ingroup, “the pure people,” with an antagonistic and inherently evil outgroup, “the corrupt elite.” This ingroup of “pure people” allows almost everyone to self-assign, due to its high social inclusiveness. At least, this is true for people who do not violate (implicit or explicit) group boundaries of populist ideology often defined by ethnic, socioeconomic, or opinion-based criteria. At the same time, populist ideology may create a sense of collective agency, or control (Fritsche, 2022), by displaying the ingroup (“the people”) as having joint goals, pursuing goals, and being likely to attain them: First, populism assumes a “general will” and the “sovereignty” of the people (Mudde & Kaltwasser, 2017; Wuttke et al., 2020), thus ascribing *autonomous joint ingroup goals*. Second, according to populist reasoning, collective *goal directed action* seems not just legitimate but even required, given the proposed fight against the “corrupt elite” as an antagonistic outgroup who is in opposition to the natural interests of the ordinary people (Mudde, 2004; Vehrkamp & Merkel, 2019) and incorporates the absolute evil (Manichean outlook; Wuttke et al., 2020). Third, the populist idea of “sovereignty of the people,” for example, through plebiscite (Mudde & Kaltwasser, 2017; Wuttke et al., 2020), implies that “the people” can indeed have a direct impact on their environment (*collective efficacy*). Taken together, we assume that adopting populist attitudes bolsters people’s sense of control through their collective and, thus, personal, self.

There is first evidence for a control function of populist attitudes in correlational research indicating that lack of personal control might increase people’s support for radical populist parties of both the left and the right political spectrum (Heinisch & Jansesberger, 2024; Spruyt et al., 2016). Subsequently, adopting populist attitudes may increase people’s sense of control. However, there has been no systematic causal testing of populism effects on perceived control, yet. We address this gap in the present research.

## The Present Research

We straightforwardly test the idea that populist attitudes (here based on the definition developed by Mudde & Kaltwasser, 2017) increase peoples’ sense of personal control. Specifically, we conducted three experiments to examine whether populist attitudes increase people’s perceptions of control regarding different objects of control (i.e., control over the immediate situation, over social discourse, over life in general) and different representations of the self (i.e., personal and collective self). Therefore, we developed a method to manipulate populist attitudes by either inducing a populist or nonpopulist mindset in individuals. Participants were asked to prepare for an upcoming debate (Studies 1 and 2) or to design creative slogans (Study 3) in a populist or nonpopulist fashion, depending on the experimental condition. With this method, we relied on self-perception theory which proposes, that people derive their attitudes from their own behavior (Bem, 1972) and that the accessibility of attitudes guides subsequent perceptions and behavior (Fazio & Williams, 1986). Previous studies testing this idea showed, for instance, that women’s speaking time in a leadership task was a precursor of their self-evaluated leadership performance (Latu et al., 2013). Self-perceived travel behavior predicted personal attitudes toward tourism (Woosnam et al., 2018), and adherence to organizational COVID-19 guidelines was a precursor of employees attitudes toward COVID-19 vaccination (Roswag et al., 2023). Concerning the current manipulation of populist attitudes, we chose a topic (raising the retirement age) where it was easy for participants to position themselves against it. Also, the participants probably have not given much thought to this issue beforehand. It therefore seemed reasonable that the engagement with self-developed arguments had an impact on the participants’ attitudes and put them in a state of either thinking in a populist or nonpopulist way. After manipulating populist attitudes (and additionally control salience in Study 3), we measured control perceptions.

As a control group, we decided for inducing pluralist attitudes, which is typically seen as counterpart of populism (Mudde & Kaltwasser, 2017; Müller, 2017; Priester, 2007; Vehrkamp & Merkel, 2019). While populist ideology describes “the people” as homogeneous entity holding identical values, interests, and goals (Mudde, 2004), pluralism holds that a society is divided into a broad variety of partially overlapping social groups with different ideas and interests. Diversity is seen as strength. Politics is valued for its capacity to achieve compromise and consensus, reflecting the need to address the divergent interests and values of many societal groups (Mudde & Kaltwasser, 2017).

## Study 1

In Study 1, we experimentally manipulated populist attitudes by asking online participants to prepare either a populist or pluralistic reasoning for an upcoming online debate. After the manipulation, we measured control perceptions. The study was preregistered (aspredicted.org, #74101).

### Method

Materials, analysis code, data, codebook, and the preregistrations of all studies are available can be found here: https://osf.io/36je2/?view_only=829feeb57f984a8c852fa666ae5320bc. Deviations from the preregistrations and correlation tables for all studies and distributions of core variables can be found in the supplementary materials. All studies, measures, manipulations, and data/participant exclusions are reported in the manuscript.

#### Participants and Design

We used a one-factorial design. A priori, we calculated a required sample size of *N* = 128 by using G*Power (Faul et al., 2007), planning to perform two-sided *t* tests that should be able to detect a medium effect (*d* = .50) with a power of .80, and an α error of <.05. We recruited 128 participants online (e.g., via sharing the link in Facebook groups). As an incentive, three online shopping vouchers (1x€50, 2x€25) were raffled off. As two participants did not sign informed consent, the final sample included 126 participants (77 women, 43 men, one non-binary, five participants not indicating their gender) with a mean age of *M* = 25.48 (*SD* = 6.17).

#### Procedure and Materials

After giving informed consent, participants were asked to take part in a short online debate, ensuing later on. In addition, they were told,

To simulate a controlled debate, we will assign you to a certain opinion and a certain way of arguing that you are asked to adopt in the debate. You can think of this task as participating in a debate club, where such an assignment is common.

Next, participants received the information that the debate would be about raising the retirement age to 75 years (the retirement age in Germany at that time was 67). Afterward, all participants read a short bogus newspaper article created for this study, stating there currently was a public debate on raising retirement age to 75, and providing them with some possible pro or contra arguments. Afterward, *all* participants were informed that they were randomly assigned to argue *against* raising the retirement age in the subsequent debate and asked to take some time to prepare their arguments. For the manipulation of populist attitudes, participants were randomly assigned to one of two different conditions. In the populist condition, based on the populism definition by Mudde and Kaltwasser (2017), participants were asked to prepare specific arguments that represent each of the following elements of reasoning. (a) Their arguments represent the will of the people in their country, which drives their behavior; (b) their position represents the true interests of the people in the country, which were disregarded by a corrupt elite; (c) their position is good, contrary positions are bad; and (d) that the people in the country are in complete agreement about this position. In the nonpopulist condition, participants were asked to prepare their arguments in line with the following elements, representing pluralistic reasoning. (a) Their arguments represent *personal* interests and points of view that are not yet put into action; (b) there are *other* opinions within society, as well; (c) their position reflects *personal* evaluations and attitudes; and (d) their position reflects certain interests whereas the contrary position reflects others. Then, in both conditions, participants were asked to write down their arguments in an open text box and to tick off each of the four elements of reasoning that they had already considered.

Then, we measured participants’ perception of control (29 items in total; ω = .93) using four subscales representing different objects of control and representations of the self (i.e., personal or collective self). All of these measures reflect the three indicators of control (Fritsche, 2022; Stollberg et al., 2015), namely whether participants had own goals, acted toward their goals, and felt able to achieve them. We randomized item order within each subscale and used 5-point rating scales from 1 (*strongly disagree*) to 5 (*completely agree*).

The first subscale measured respondents’ anticipated control in the upcoming debate, using 14 items (ω = .91, e.g., “For the upcoming debate, I myself have very clear goals in mind.,” “In the upcoming debate, I will actively defend my position,” “In the upcoming debate, I will achieve my goals.”). A full list of all items for all studies can be found in the supplementary materials. Then, we measured participants perceived personal control over the societal retirement discourse by four items (ω = .74, e.g., “I am able to personally contribute to the societal discussion on retirement at 75 in everyday life,” “I am certain that I personally have an influence on whether retirement at 75 actually comes in the end.”). Control over life in general was then measured by six items, including three self-generated items and three items based on Greenaway et al. (2013) (ω = .87, e.g., “In general, I actively work toward my goals in life,” “I feel in control of my life”). Finally, we included a subscale on how much control participants attributed to their opinion-based ingroup, using five items (ω = .85, e.g., “The group of people who are against retirement at 75 are actively working together,” “. . . can achieve their goals”).

After measuring perceptions of control, we assessed whether participants felt comfortable with their way to prepare for the debate (ω = .76), using three items (e.g., “The way I am supposed to argue corresponds to my personal way of arguing.”). Then, we measured sociodemographic data and participants’ motivation to prepare for the upcoming debate as well as comprehensibility of, and compliance with the instructions. Furthermore, we measured whether the instructions triggered contradiction in them and how much effort participants put into filling out the questionnaire.

At the end, participants were shown their written notes again, which they had prepared for the debate to check for the manipulation’s effectiveness. As a manipulation check, participants were asked how much their own arguments represented eight different points of reasoning with four items representing the criteria of populist ideology (ω = .81, e.g., “Your position represents the true interests of the people in the country, which are, however, disregarded by a corrupt elite.”) and four items representing the criteria of nonpopulist, pluralistic attitudes (ω = .72, e.g., “Your position represents personal interests that have not yet been put into action.”).

Finally, participants were fully debriefed, and we explained that there would be no debate. Before again requesting informed consent, we asked participants to rate the extent to which they had assumed that a debate would take place. For exploratory purposes and after our central dependent measures we additionally measured self-esteem (two items), certainty (6), populist attitudes (10), previous engagement with the topic of retirement, attitudes about retirement age, ingroup identification, personal relevance of the topic of retirement, and excitement before the upcoming debate (one item each). These analyses are not part of this article. Details on results can be obtained from the first author. Data were analyzed with the open-source software Jamovi (The Jamovi Project, 2024). Distributions of core variables are part of the supplementary materials (Figures S1, S2, and S3).

### Results

Full statistics are included in the notes and are linked with superscript numbers.

#### Manipulation Check and Credibility of the Upcoming Debate

As a manipulation check, we tested whether the manipulation increased participants’ self-assessment of how much their reasoning was characterized by the four populism criteria (composite measure). Indeed, a t-test revealed higher populist reasoning in the populist condition (*M* = 3.36, *SD* = 0.92) compared with the nonpopulist condition (*M* = 2.81, *SD* = 0.86), *t*(119) = −3.40, *p* < .001, *Cohen’s d* = −0.62, confidence interval (CI) = [−0.98, −0.25].

Next, we tested whether participants’ arguments met nonpopulist criteria. Here, values were descriptively higher in the nonpopulist condition (*M* = 3.58, *SD* = 0.80) compared with the populist condition (*M* = 3.30, *SD* = 0.84), *t*(119) = 1.86, *p* = .065, *Cohen’s d* = 0.34, CI = [−0.02, 0.70].

We additionally analyzed if participants were convinced that a debate would take place. Overall, credibility ratings were only moderate (*M* = 2.33, *SD* = 1.19, on a scale from 1 to 5), but did not differ significantly between the two conditions, *t*(124) = −0.50, *p* = .620, *Cohen’s d* = −0.09, CI = [−0.44, 0.26]. Differences in degrees of freedom are due to missing values.

#### Differences in Feeling Comfortable with the Way of Arguing

A *t*-test revealed that participants in the nonpopulist condition (*M* = 3.35, *SD* = 0.73) felt significantly more comfortable with their assigned reasoning compared with participants in the populist condition (*M* = 2.76, *SD* = 1.15), *t*(118) = 3.33, *p* = .001, *Cohen’s d* = 0.61, CI = [0.24, 0.98].

#### Differences in Control Perceptions

We tested our main hypothesis that induced populist attitudes increased participants’ perceptions of control by conducting ANCOVAS, first for a composite measure of control and then for each of the four subscales of perceived control over the debate, over the societal retirement discourse, over life in general and through the ingroup. As, unexpectedly, people in the populism (vs. the nonpopulism) condition felt less comfortable with their own reasoning, we included personal comfortableness as a covariate in all analyses, thus deviating from the preregistered use of simple *t*-tests.

**Figure 1.**
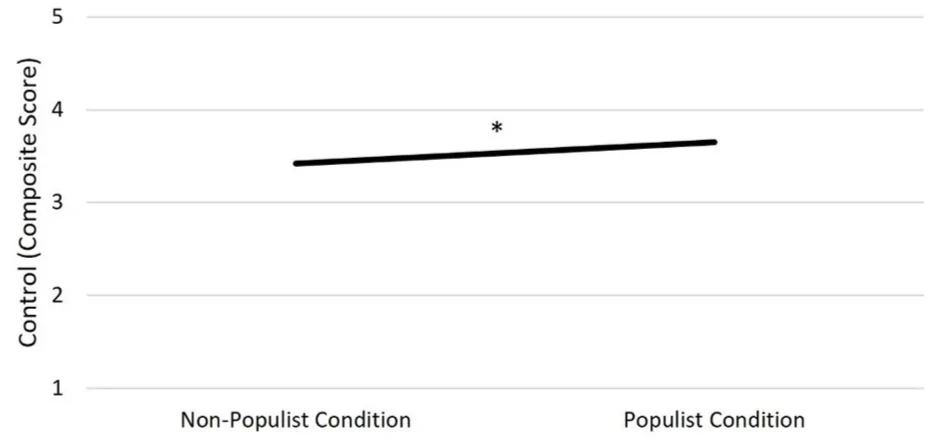
Effects of the Populist Attitudes Manipulation on Control Perceptions (composite score), Controlling for Feeling Comfortable With Induced Reasoning in Study 1 (*p < .05).

As predicted, overall control scores (estimated marginal means) were significantly higher in the populist (*M* = 3.65, *se* = 0.07) compared to the nonpopulist condition (*M* = 3.42, *se* = 0.07; see Figure 1), *F*(117) = 5.22, *p* = .024, *Cohen’s d* = −0.44, CI = [−0.82, −0.05]. The sample size of *N* = 120 participants provided 80% power to detect an effect size of *Cohen’s d* = 0.25 or greater in an ANCOVA with one independent variable (two conditions) and one covariate with a 5% false-positive rate.

**Figure 2.**
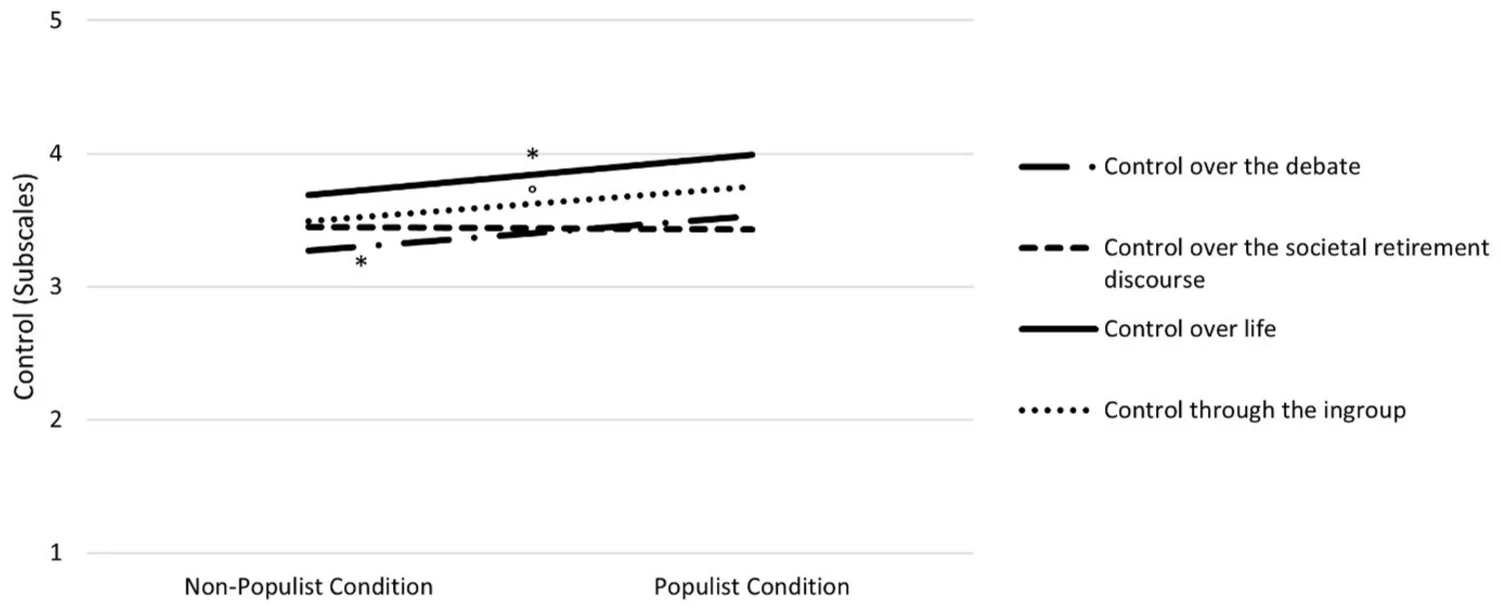
Effects of the Populist Attitudes Manipulation on the Control Subscales, Controlling for Feeling Comfortable with Induced Reasoning in Study 1 (°*p* < .10; *p < .05).

On subscales level (see Figure 2), the populism (vs. nonpopulism) condition was associated with significantly higher scores on perceived control over the debate (*M* = 3.53, *se* = 0.08 vs. *M* = 3.27, *se* = 0.09), *F*(117) = 4.66, *p* = .033, *Cohen’s d* = −0.41, CI = [−0.80, −0.03]. The same applied to perceived control over life (*M* = 3.99, *se* = 0.09 vs. *M* = 3.69, *se* = 0.10), *F*(117) = 4.73, *p* = .032, *Cohen’s d* = −0.42, CI = [−0.80, −0.03]. Descriptively, we found higher scores for perceived control through the ingroup (*M* = 3.75, *se* = 0.10 vs. *M* = 3.49, *se* = 0.10), *F*(117) = 3.12, *p* = .080, *Cohen’s d* = −0.34, CI = [−0.72, 0.04].

Conditions did not significantly differ concerning perceived control over the societal retirement discourse (*M* = 3.43, *se* = 0.10 vs. *M* = 3.45, *se* = 0.11), *F*(117) = 0.01, *p* = .925, *Cohen’s d* = 0.18, CI = [0.36, 0.40]. It should be noted that none of the preregistered *t*-tests not including feeling comfortable as a covariate, reached statistical significance (all *p* > .12). However, descriptive trends are similar (except for a reversed but nonsignificant trend for the topic of retirement as dependent variable, *p* = .224^1^).

### Discussion

We found first causal evidence that populist attitudes increase people’s perceptions of control. Participants who prepared in a populist (vs. nonpopulist) way for an upcoming debate perceived higher control (composite score, control over the debate, life in general and, descriptively, through the ingroup).

Nevertheless, we did not find any effect on the belief to control the societal discourse on retirement. Possibly, several people did not believe that the debate would take place and might not have invested too much effort in preparing arguments, increasing the doubt to convince people in everyday contexts. Manipulation check analyses demonstrated that the manipulation elicited populist reasoning, but we found only descriptive effects on nonpopulist reasoning. Possibly, the low credibility of the experimental scenario reduced manipulation strength. Raising experimental reality could intensify the effects which we aimed at in Study 2.

## Study 2

Study 2 served to replicate the findings of Study 1. We intended to enhance participants’ belief that they would take part in a real debate to intensify the manipulation. Study 2 was conducted in the laboratory and preregistered (aspredicted.org, #101659).

### Method

#### Participants and Design

A-priori power analysis using G*Power resulted in a required sample size of *N* = 194, given an estimated effect size of *d* = .047 (Study 1 effect on control over life when excluding participants who did not believe that the debate would take place), an aspired α error of <.05 (for the initially planned *t*-tests), and power of .90. Adding some oversampling, this led to a planned sample size of 210 participants. Thus, we stopped recruiting after having gathered 211 participants (122 women, 84 men, 1 non-binary and 4 not indicating their gender) with a mean age of *M* = 22.73 (*SD* = 4.70).

#### Procedure and Materials

Study 2 was conducted in the laboratory. First, participants were contacted by a student assistant at a local university campus and asked whether they would like to participate in a 30-min study about debating. Those who agreed were guided to the laboratory where several rooms on the floor were labeled “debate room” to enhance the participants’ belief that they would take part in a real debate.

Upon arrival, participants were welcomed, seated at an individual laptop computer (workplaces separated with partitions), instructed to be quiet and avoid talking to other participants, and asked for informed consent. They received a personal ID number, which they were asked to transfer to a blank sheet provided for personal notes and to enter in the electronic questionnaire.

Similar to Study 1, participants were asked to prepare for a 10- to 15-min debate and they were told that they would be assigned a specific opinion and way of arguing which they had to adopt in the debate and for the preparation of the discussion. They also received the information that the debate would be on the topic of “retirement at age 75” and read the bogus newspaper article about raising the retirement age used in Study 1.

Afterward, participants were randomly assigned to one of the two experimental conditions. The instructions were identical to Study 1. The only difference was that participants handwrote their preparatory notes (about their planned reasoning) on the blank sheet.

Afterward, we measured perceived control by 21 items (ω = .82) representing the four facets of control we measured in Study 1 (control over the debate, over the societal retirement discourse, over life, and through the opinion-based ingroup). The subscales were presented in this order. Within the subscales, items were presented in randomized order. This time, we used 7-point response scales from 1 (*not true at all*) to 7 (*fully true*) (instead of 5-point scales in Study 1).

Control over the upcoming debate was measured with six items (ω = .76; due to economic reasons we decided to reduce this subscale compared with Study 1 (see supplementary materials). The other control subscales were identical to those of Study 1: Control over the societal retirement discourse (four items, ω = .58), control over life (six items, ω = .83), and control through the ingroup (five items, ω = .72).

Afterward, we assessed whether participants felt comfortable with their way of arguing by using four items (ω = .85). Items 1 to 3 were identical to Study 1. We added the following item: “It is easy for me to get in touch with this way of arguing.” This was followed by the manipulation check, which was identical to Study 1 (ω = .82 for the populist criteria, ω = .68 for the nonpopulist criteria). This time, we used 7-point scales from 1 (*not true at all*) to 7 (*fully true*).

Afterward, we assessed sociodemographic information, asked whether participants feared to be affected by poverty in old age, their political attitudes and voting behavior, if they have already participated in a similar study and how much effort they put in filling out the questionnaire. Once participants had completed the questionnaire, they were taken to another room. There, they were informed that there would be no debate taking place. They received an incentive (5 Euro), the debriefing, and another questionnaire where we assessed their previous belief that the debate would take place and what they thought the goal of the study would be.

For exploratory purposes and after we measured control perceptions, the questionnaire additionally included other measures. We captured populist attitudes (10 items), ingroup identification (5), attitudes about retirement age, excitement before the upcoming debate, the belief to persuade one’s discussion partner, whether debate preparation gave participants the opportunity to deal with counter-positions, whether they felt prepared, gained knowledge about retirement, and had the feeling that their opinion is legitimate (one item each). Analyses on these variables are not part of this article. Details can be obtained from the first author.

### Results

#### Manipulation Check and Credibility of the Upcoming Debate

Supporting the effectiveness of the populism manipulation, participants more strongly rated their prepared arguments as representing populism criteria in the populist condition (*M* = 4.67, *SD* = 1.51) compared with the nonpopulist condition (*M* = 3.60, *SD* = 1.09), *t*(209) = −5.91, *p* < .001, *Cohen’s d* = −0.81, CI = [−1.10, −0.52]. Also, as expected, for ratings of *nonpopulist* criteria the pattern was reversed (*M* = 4.38, *SD* = 1.14 in the populist condition; *M* = 5.17, *SD* = 0.95 in the nonpopulist condition), *t*(209) = 5.45, *p* < .001, *Cohen’s d* = 0.75, CI = [0.46, 1.04].

We managed to markedly improve the credibility of the cover story compared with Study 1, as participants were highly convinced that a debate would take place (*M* = 6.25, *SD* = 1.21, on a scale from 1 to 7) and this conviction did not differ significantly between the two conditions, *t*(207) = 0.78, *p* = .434, *Cohen’s d* = 0.11, CI = [−0.16, 0.38].

#### Differences in Feeling Comfortable With the Way of Arguing

As in Study 1, participants in the nonpopulist condition (*M* = 4.56, *SD* = 1.36) felt significantly more comfortable with their way of arguing than participants in the populist condition (*M* = 3.82, *SD* = 1.53), *t*(209) = 3.71, *p* < .001, *Cohen’s d* = 0.51, CI = [0.23, 0.79].

#### Effects on Perceptions of Control

**Figure 3.**
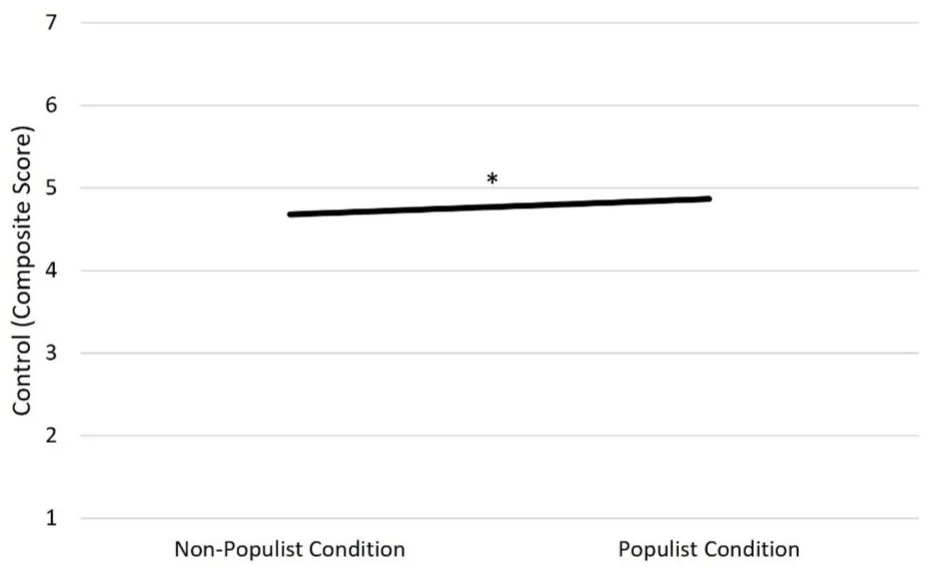
Effects of the Populist Attitudes Manipulation on Control Perceptions (composite score), Controlling for Feeling Comfortable With Induced Reasoning in Study 2 (*p < .05).

As in Study 1, we tested our hypothesis, that populist attitudes increase people’s sense of control by using ANCOVAS with feeling comfortable with the manipulation included as a covariate. Regarding the composite measure of control (Figure 3), estimated marginal means were higher in the populist (*M* = 4.87, *se* = 0.06) compared with the nonpopulist condition (*M* = 4.68, *se* = 0.06), *F*(208) = 4.88, *p* = .028, *Cohen’s d* = −0.31, CI = [−0.60, −0.03]. The sample size of *N* = 211 participants provided 80% power to detect an effect size of *Cohen’s d* = 0.19 or greater in an ANCOVA with one independent variable (two conditions) and one covariate with a 5% false-positive rate.

**Figure 4.**
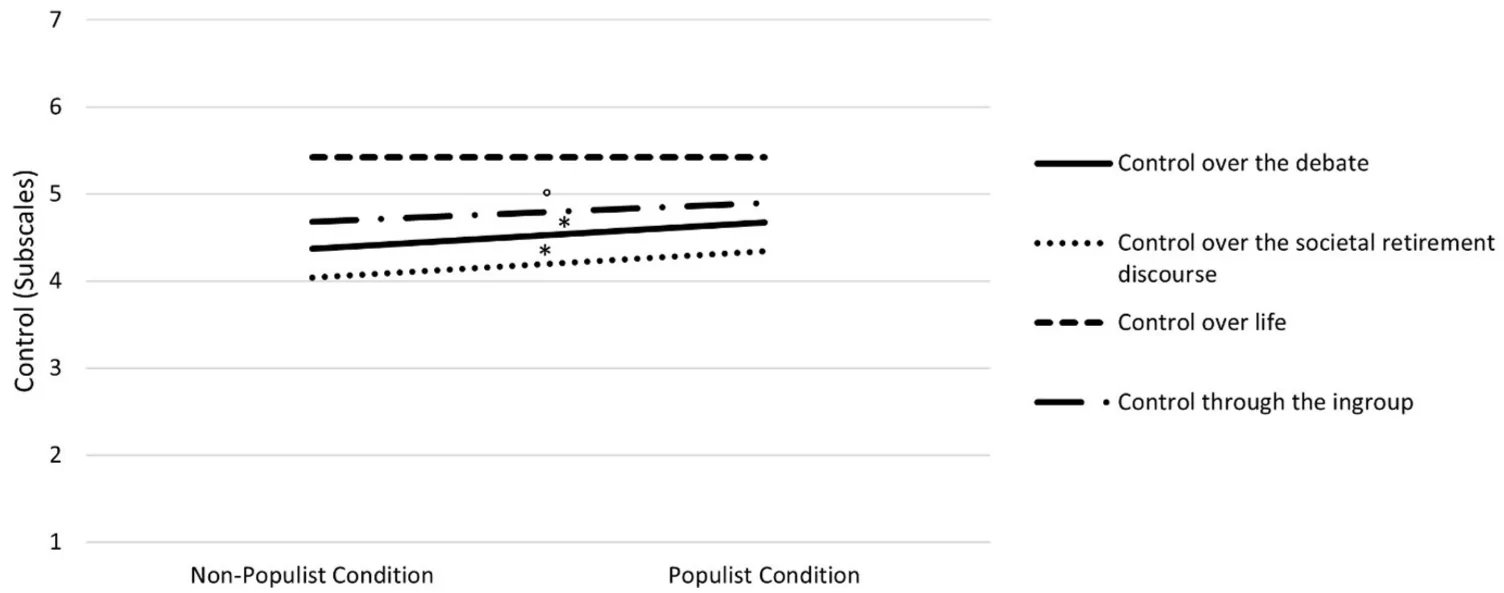
Effects of Populist Attitudes Manipulation on the Control Subscales, Controlling for Feeling Comfortable with Induced Reasoning in Study 2 (°*p* < .10; *p < .05).

Analyses on the subscales (Figure 4) revealed significantly higher control scores in the populist versus nonpopulist condition for perceived control over the debate (*M* = 4.67, *se* = 0.09 vs. *M* = 4.37, *se* = 0.09), *F*(208) = 4.92, *p* = .028, *Cohen’s d* = −0.32, CI = [−0.60, −0.03]. The same applied to control over the societal retirement discourse (*M* = 4.34, *se* = 0.10 vs. *M* = 4.04, *se* = 0.10), *F*(208) = 4.22, *p* = .041, *Cohen’s d* = −0.29, CI = [−0.57, −0.01], and control through the ingroup (*M* = 4.90, *se* = 0.09 vs. *M* = 4.68, *se* = 0.09), *F*(208) = 2.87, *p* = .092, *Cohen’s d* = −0.24, CI = [−0.52, 0.04], only descriptive tendencies.

In contrast to Study 1, control over life did not differ between conditions (*M* = 5.42, *se* = 0.09 vs. *M* = 5.42, *se* = 0.09), *F*(208) = 0.00, *p* = .991, *Cohen’s d* = −0.00, CI = [−0.28, 0.28]. Not including feeling comfortable as a covariate led to nonsignificant results for all respective *t*-tests (all *p* > .28^2^). However, descriptive tendencies remain similar.

### Discussion

Again, the induction of populist thinking increased people’s sense of control. The manipulation check was successful for both populist and nonpopulist arguments. Compared with Study 1, participants were more convinced that they would take part in a debate which might have led them to work more intensely on their arguments. Therefore, they also felt more prepared to exert control on the social retirement discourse. At the same time, participants’ strong goal focus may have prevented an effect on control over life *in general*. Alternatively, the missing effect might be due to a ceiling effect, as the perception of control over life was quite high compared with the other subscales.

In Studies 1 and 2, participants in the populism condition felt less comfortable with their assigned reasoning style, which was included as a covariate (in deviation from the preregistration). This might have been due to their expectation to meet a discussion partner who potentially rejects populist arguing. Therefore, in Study 3, we provided a context without interpersonal contact to be expected.

## Study 3

Study 3 served the replication of the previous findings in an anonymous online context. We asked participants to prepare creative populist versus nonpopulist slogans. In addition, we were interested in whether salient threat to personal control would strengthen the populism effect on perceived control (we preregistered this additional hypothesis; see aspredicted.org, #117193).

### Method

#### Participants and Design

A priori, we calculated a required sample size of *N* = 400 (using G*Power), planning to perform an analysis of variance (ANOVA) with two factors (*f* = .15, power = .80, alpha = .05, some oversampling). Finally, the sample contained 401 participants recruited on the campuses of two German Universities (*N* = 250) and online via the micro-task portal clickworker.com (*N* = 151). As an incentive, one voucher á €50 was raffled off. Five people did not give consent to data processing. Thus, the final sample contained 396 participants—216 female, 167 male, 10 non-binary, three not indicating their gender, mean age *M* = 31.35 (*SD* = 12.18).

#### Procedure and Materials

After obtaining informed consent, we assessed demographic data. Then, to manipulate low vs. high control salience, participants were asked to recall, and then describe, a positive event (not) caused by themselves and which they could (not) influence, depending on condition (Kay et al., 2008). This was followed by a proximal manipulation-check item, asking participants to rate whether this event involved *very low* (1) to *very high* (7) *control*.

Then, as a cover story, participants were told they had to develop creative slogans that would be used in future studies and would then be rated according to several criteria. Afterward, the procedure was similar to Studies 1 and 2. Participants read the bogus newspaper article on raising retirement age and were asked to argue against raising the retirement age. Then, they were randomly assigned either to the populist or nonpopulist instruction of how to argue in their slogans. Subsequently, participants were asked whether they enjoyed the task and whether they found it easy on 7-point Likert-type scale from 1 (*don’t agree at all*) to 7 (*completely agree*).

Afterward, we measured perceived control as in Study 2 (21 items, ω = .88). The only difference was that the first subscale measured control through one’s slogans instead of control over the debate in Studies 1 and 2 (e.g., “My slogans will convince everyone of my position.,” ω = .78). The other control subscales were identical to Studies 1 and 2, control over the societal retirement discourse (ω = .73), over life (ω = .87), and through the ingroup (ω = .83). Items were randomized within the subscales and answered on 7-point scale from 1 (*not true at all*) to 7 (*fully true*).

As in Studies 1 and 2, participants were asked whether they felt comfortable when designing the slogans. We used the same four items as in Study 2 but adapted to the slogans (e.g., “It was easy for me to integrate the given aspects into the slogans.”; ω = .67). The populist attitudes manipulation-check items were identical to the previous studies (ω_populist_ = .85, ω_nonpopulist_ = .67).

At the end of the questionnaire, participants were asked whether they had already participated in similar studies, how much effort they put in filling out the questionnaire, and whether they believed that their slogans would be used in future studies. For exploratory reasons and after our central dependent measures we also added items to measure behavioral intentions (3), ingroup identification (5), attitudes against raising the retirement age (1), populist attitudes (10), threat of old-age poverty (1), political orientation (1), and party choice (1). Analyses on these variables are not part of this article. Details can be obtained from the first author.

### Results

#### Manipulation Checks and Credibility of Slogan Design

Supporting the effectiveness of the control manipulation, participants attributed less control to their described event in the low (*M* = 2.39, *SD* = 1.52) compared with the high control condition, *M* = 5.63, *SD* = 1.30; *t*(394) = −22.80, *p* < .001, *Cohen’s d* = −2.29, CI = [−2.55, −2.04].

The results of two 2 (high/low control salient) × 2 (populist/nonpopulist reasoning) ANOVAs indicated the effectiveness of the populism manipulation. Participants in the populist condition more strongly rated their slogans as representing populism criteria (*M* = 4.63, *se* = 0.10) compared with those in the nonpopulist condition, *M* = 3.95, *se* = 0.10; *F*(1, 392) = 24.62, *p* < .001, η_p_^2^ = .06, CI = [.02, .11]. As expected, we found a reversed pattern for the nonpopulist criteria with higher values in the nonpopulist (*M* = 5.22, *se* = 0.07) compared with the populist condition, *M* = 4.86, *se* = 0.07; *F*(1, 392) = 13.73, *p* < .001, η_p_^2^ = .03, CI = [.01, .08]. No other effects were found (all *p* > .10^3^).

The credibility of the cover story was acceptable (*M* = 4.01, *SD* = 1.81, on a scale from 1 to 7). The ratings were not affected by the manipulations of control salience and populist attitudes, and we did not find any interaction effects (all *p* > .27^4^).

#### Differences in Feeling Comfortable with the Way of Slogan Design

Feeling comfortable was not affected by the manipulations (all *p* > .18^5^).

#### Effects on Perceptions of Control

We tested the effects of control salience and populist reasoning on control perceptions, employing ANCOVAS including “feeling comfortable” as a covariate (for comparability with Studies 1 and 2). In addition, we present ANOVAs without covariate, as well.

**Figure 5.**
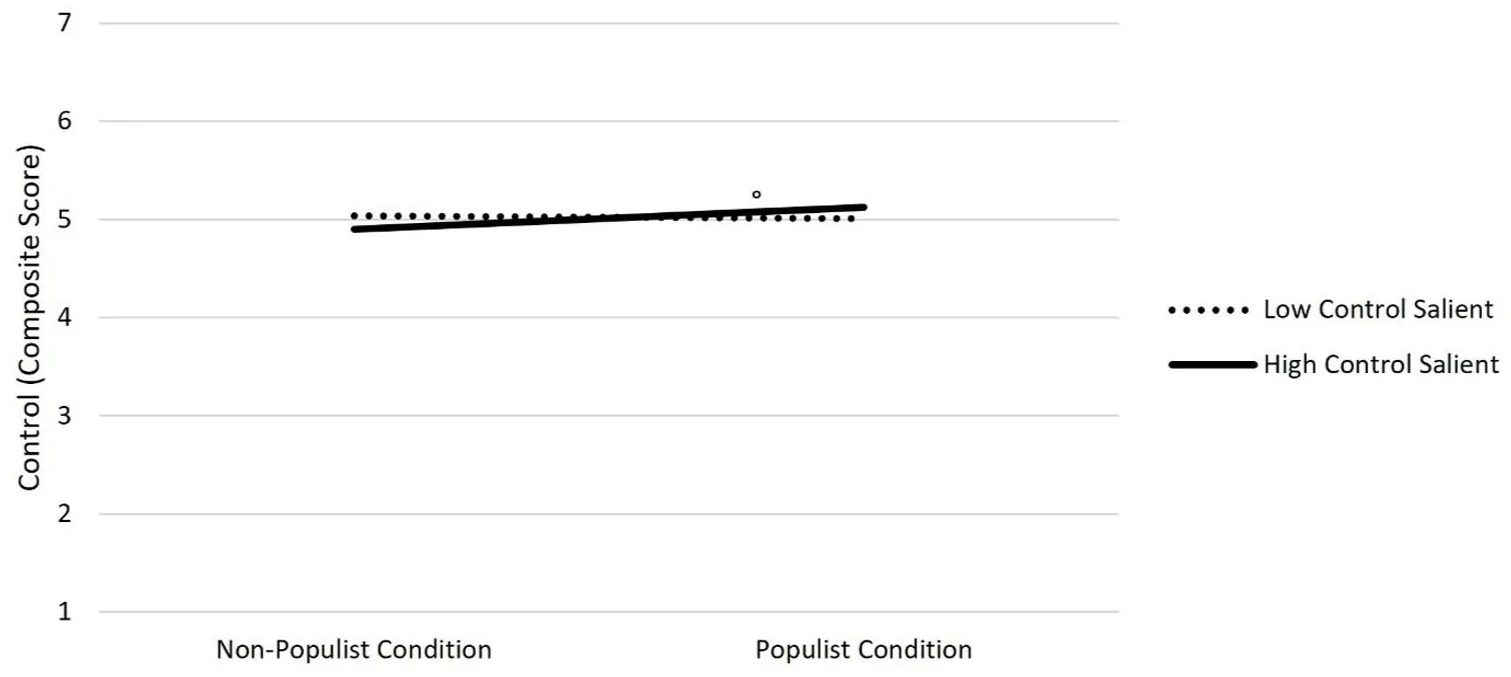
Effects of Control Salience and Populist Attitudes on Overall Control Perceptions, Controlling for Feeling Comfortable With Induced Reasoning in Study 3 (°*p* < .10).

Regarding the composite control measure, we did not find main effects of control salience, *F*(1,391) = 0.10, *p* = .752, η_p_^2^ = .00, CI = [.00, .01], or populist attitudes, *F*(1, 391) = 2.02, *p* = .156, η_p_^2^ = .01, CI = [.00, .03], but indications of an interaction of both variables, *F*(1, 391) = 3.54, *p* = .061, η_p_^2^ = .01, CI = [.00, .04] (see Figure 5). Post hoc tests (Tukey-corrected) reveal that when high control was salient, participants in the populism condition (*M* = 5.13, *se* = 0.06) reported descriptively higher control compared with the nonpopulism condition, *M* = 4.90, *se* = 0.07; *t*(391) = −2.37, *p* = .085, *Cohen’s d* = −0.33, CI = [−0.61, −0.06]. No difference between populism conditions occurred when low control was salient, *M* = 5.01, *se* = 0.07 versus *M* = 5.04, *se* = 0.07; *t*(391) = 0.32, *p* = .99, *Cohen’s d* = 0.05, CI = [−0.24, 0.33], all other *p*s > .40^6^. When the covariate is *not* included, we find a significant interaction of both independent variables, *F*(1,392) = 3.92, *p* = .048, η_p_^2^ = .01, CI = [0.00, 0.04]. However, post hoc *t* tests do not reveal significant differences (all *p*s > .10^7^). Referring to the ANOVA, the sample size of *N* = 396 participants provided 80% power to detect an effect size of η_p_^2^ = .027 or greater in a two-factorial ANOVA (2 × 2 design) with a 5% false-positive rate.

**Figure 6.**
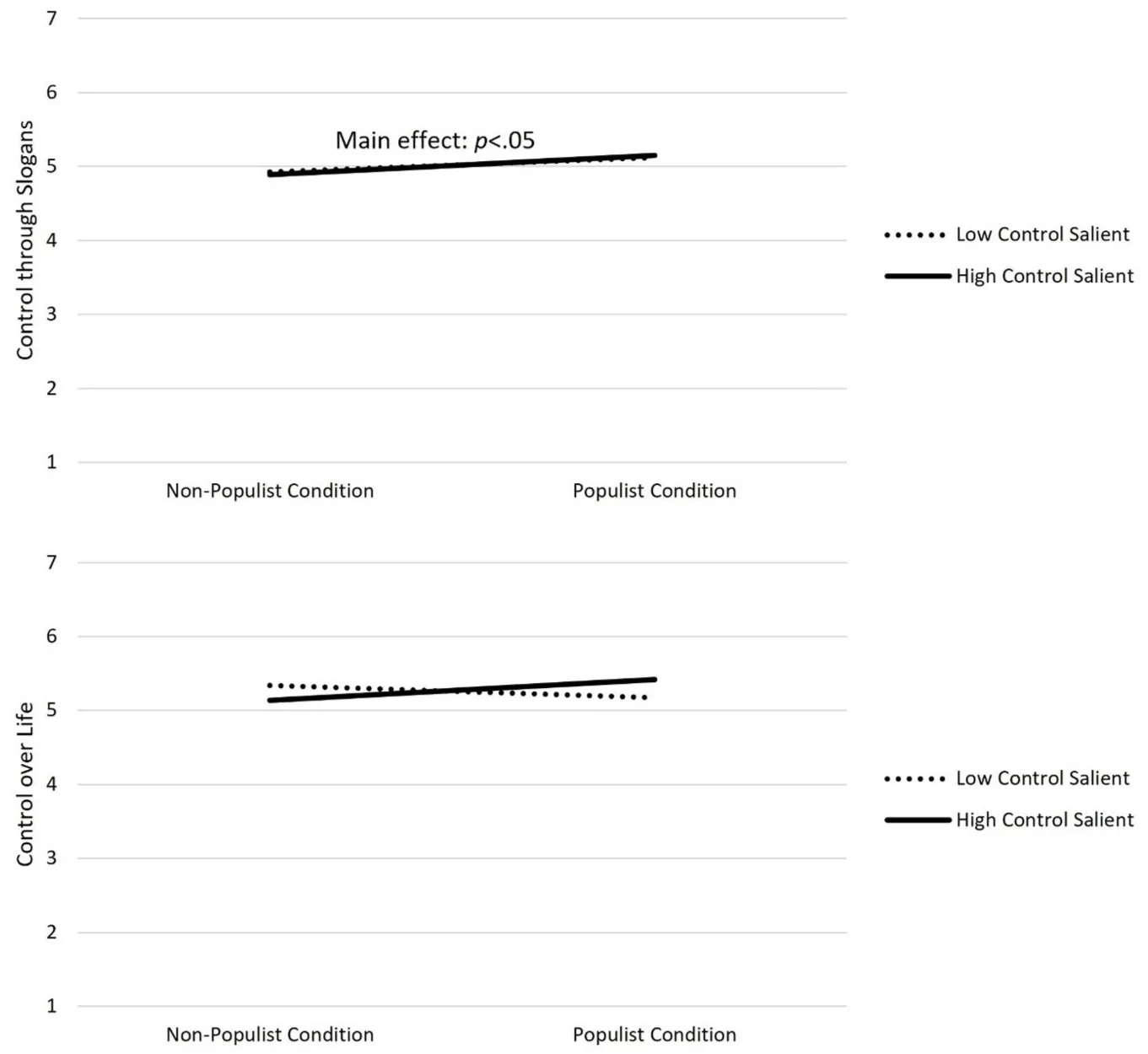
Effects of Control Salience and Populist Attitudes on (a) Control through slogans and (b) Control Over Life, Controlling for Feeling Comfortable With Induced Reasoning in Study 3.

Regarding the subscale control through one’s slogans, the ANCOVA reveals a main effect of populist attitudes, *F*(1,391) = 7.31, *p* = .007, η_p_^2^ = .02, CI = [.00, .05] (see Figure 6). Estimated marginal means are higher in the populist (*M* = 5.14, *se* = 0.06) compared to the nonpopulist condition (*M* = 4.91, *se* = 0.06). No other effects were significant (both *p* > .688). The pattern is similar if the covariate is *not* included, with a main effect of populist attitudes, *F*(1,392) = 5.30, *p* = .022, η_p_^2^ = .01, CI = [.00, .04].^9^

Regarding the subscale control over life, the ANCOVA reveals a significant interaction of control and populist attitudes, *F*(1,391) = 5.47, *p* = .020, η_p_^2^ = .01, CI = [.00, .05] (Figure 6). Tukey corrected post hoc comparisons do not reveal significant simple effects (all *p* > .1510). No main effects were present (*p*s > .5611). The interaction effect is similar when the covariate is *not* included (*p* = .016, η_p_^2^ = .02, CI = [.00, .05]^12^).

No effects were found for the subscales “control over the societal retirement discourse” (*p*s > .17)^13^ and “control through the ingroup” (all *p* > .21)^14^.

### Discussion

Study 3 conceptually replicates the finding that inducing populist attitudes increases people’s sense of control, at least concerning their immediate goals (here: affecting others by own political slogans). At the same time, the effect on perceived control over life seemed to be eliminated when people were reminded of low (vs. high) control. This contradicts our preregistered hypothesis that threat to control would elevate the effect. As a post hoc explanation, we assume that induced salience of low control may have lead people to motivationally bias their control perceptions upward (Kay et al., 2008, Study 2). This should apply to the nonpopulist condition but not when participants were allowed to act in a populist manner. Populist reasoning might have worked to elevate people’s sense of situational control, thus relieving participants from the need to exaggerate global control perceptions after salient control threat. Second, salience of low control may have reduced the effectiveness of the subsequent manipulation of populist attitudes to increase participants’ control beliefs. This is because it should have shattered all participants’ sense of control over their own life (the measure for which the interaction pattern was most pronounced).

In Study 3, participants felt equally comfortable with their reasoning. The fact that we obtain similar effects when not controlling for comfortableness, strengthens our trust in the robustness of the basic effect of populism on control.

## General Discussion

What is the motivational benefit of populist thinking? We tested a novel group-based control account to answer this question. Specifically, we proposed that populist attitudes provide people with a sense of control through their (social) self. Following the definition by Mudde and Kaltwasser (2017), populism conceives of individuals as being part of a large and forceful group (“the people”) that is legitimately called to impose their will on an antagonistic enemy outgroup (“the corrupt elite”), thus conferring subjective control through membership in an agentic group. Three experiments support our basic hypothesis that inducing populist attitudes (by inducing either populist or pluralistic reasoning) elevates people’s control perceptions. The effect was robust across studies for our primary measure of control, referring to people’s immediate goals (e.g., winning a subsequent debate). Populism priming also increased our other measures of control, such as control over societal discourse, one’s own life, and through the opinion-based ingroup, but not consistently throughout all studies and only descriptively for the latter. The present findings support group-based control theory (Fritsche, 2022) and its hypothesis that perceptions of belonging to an agentic collective (as suggested in populist ideologies) positively affects peoples’ personal sense of control (Relke et al., 2024).

This research provides first evidence that populist attitudes increase personal control beliefs. Thus, populist thinking seems useful for coping with threats to personal control. This may explain the apparent attractiveness of populist attitudes under conditions of perceived personal and societal crisis (Brubaker, 2017; Dennison & Turnbull-Dugarte, 2022; Manunta et al., 2022; Obradović et al., 2020; Spruyt et al., 2016). Future studies should examine such a specific effect of threatened personal control increasing the attractiveness of populist (vs. pluralistic) social groups. Moreover, intervention programs may counter populism by elevating people’s personal control beliefs, for instance, through empowering citizens to actively participate in democratic decision-making processes.

To our knowledge, these studies are the first that directly tested the causal effects of populism by experimentally manipulating populist attitudes. This research thus transcends the evidential value of correlational or longitudinal research. We recommend adopting this strategy in future research on the motivational underpinnings of specific political attitudes. We based our manipulation of populist reasoning on the populism definition developed by Mudde (2004), as it is well established within and beyond political science. However, the exact definition and importance of certain aspects of populism are still under discussion. For future research, our experimental paradigm is suited to investigate the effects of other forms or single aspects of populism (e.g., focusing on the antagonism toward the ‘”elite” as proposed by Katsambekis (2022).

Although populist thinking might be a viable means to restore control, participants sometimes felt uncomfortable with adopting populist reasoning when they expected to present their arguments in a subsequent debate. Thus, we statistically controlled for sense of uncomfortableness when testing populism effects in Studies 1 and 2. In Study 3, instead, where participants were not expected to present their reasoning, participants felt equally comfortable in both conditions. Thus, the public expression of populist attitudes might be suppressed in some contexts. This insight can also help to anticipate the social conditions under which populist thinking becomes likely. At public demonstrations organized by populist groups, for example, participants will not have to fear social rejection when expressing populist attitudes due to a populist social norm. Participation in such events can therefore be particularly suitable for people to (re-)gain a sense of control, which may explain why some engage in populist protests.

### Limitations

Presenting the different control measures in identical order might have been a limitation. We first measured people’s sense of control in the immediate situation (control over the upcoming debate or through the invented slogans). For this subscale, we found the most robust effect. However, due to confounding indicators and sequencing, we cannot say with certainty whether this is due to the immediacy of the control representations, or because the effect of populism simply dissipated over time. Probably, this procedure also reduced our capability to detect effects on control though one’s ingroup (descriptive effects in Studies 1 and 2 with *p* < .10; no clear pattern in Study 3, *p* > .26). According to our theorizing, populist attitudes should elevate people’s sense of control due to triggering the perception of group-based control. This is in line with previous experimental research by Jugert et al. (2016) showing repeatedly that salience of a collectively efficacious ingroup increased people’s personal efficacy beliefs via perceived collective efficacy. By placing the measure of control through the ingroup at the last position (after measuring participants’ immediate, discursive, and life control), a possible initial effect of the manipulation might have been no longer be detectable. On top of that, measuring personal control perceptions first, may have made people’s *personal* self-definition salient, thus reducing the representation of perceived control through the *collective* self, afterward. Thus, the nonsignificance of populism effects on collective control does not preclude that the manipulated populist attitudes may have elevated immediate personal control beliefs through a sense of group-based control, induced via populist thinking. Future studies should take care to place the group-based control measure immediately after the manipulation, or vary order of presentation, to detect the effects with greater reliability.

Furthermore, we did not include a neutral control group when manipulating populist vs. pluralistic reasoning. Thus, we cannot definitely say whether differences in control perceptions are due to *in*creases in the populist condition or *de*creases in the pluralistic condition. We choose this control group because first, pluralism is widely understood as the counterpart to populism (Mudde & Kaltwasser, 2017; Müller, 2017; Priester, 2007; Vehrkamp & Merkel, 2019). Second, asking participants to argue for their own position in an alternative fashion allowed us to hold participants’ active self-involvement constant across the conditions. Possibly, considering multiple concerns and perspectives, inherent in pluralist reasoning, may have reduced participants’ control perceptions. However, speaking against this alternative account, participants felt more comfortable with the induced pluralist reasoning in Studies 1 and 2. Nonetheless, thoughts about pluralism might have also made participants’ *personal* identity salient to some degree, lowering perceptions of collective control. Taken together, the question of whether the effect of populist attitudes, for example, goes beyond pure social identity salience (in the sense of a neutral control group) should be investigated in future studies.

Our experimental manipulation of populist attitudes bears some tradeoffs regarding external and internal validity. First, we only analyzed situational effects. We manipulated both, the situational cognitive accessibility of populist thinking style and its enactment, which we assume to make people adopt a populist attitude in the experimental situation (e.g., through a process of self-perception; Bem, 1972). This method made it much more likely that populist attitudes would be adopted than the mere exposure to populist speeches. Thus, the fact that we found populism activation to increase people’s immediate sense of control provides the first robust *causal* evidence that populist thinking elevates subjective control. Nonetheless, our manipulation likely did not (and should not) affect people’s chronic populist attitudes. Thus, one might question the full generalizability of our findings to the effects of chronic populism. Indeed, holding chronic populist attitudes might result in some personal attributes or social consequences that may also limit personal control (beliefs), such as advocating a minority opinion in pluralistic societies. Second, we tested for the effects of populist attitudes in the domain of retirement. This may restrict external validity regarding possible effects of ideologically enriched (left- or right-wing) populist attitudes. Future studies may transfer the manipulation to these contexts. Our manipulation checks just probed whether participants indeed produced populist content but not whether they had adopted populist attitudes, which should be done more directly in future studies. However, the latter is difficult to measure, as established populism scales (e.g., Schulz et al., 2018) typically consist of statements and terminology that can be easily identified as belonging to the discourse of specific political groups or camps, leading to social (non-)desirability bias in responding, depending on people’s personal political positioning. Also, measuring explicit populism immediately after the manipulation might make populism attitudes salient also for participants in the control condition, thus compromising the manipulation.

Finally, for testing effects of populist attitudes, it was necessary to deceive the participants about the study goal. In Studies 1 and 2, all participants expected to take part in a debate. In Study 3, they assumed to develop material for future studies. This deception served to enhance participants’ attention to the task and their self-involvement due to being actively involved in the tasks (vs. rather passive reading of information, watching populist speeches), increasing experimental impact (Wilson et al., 2010). Of course, deception should not be used without caution. It should be used as little as possible and primarily for increasing participants’ interest to provide a credible rationale for the data collection procedures (Wilson et al., 2010). After the end of the studies, all participants were fully debriefed about the real study goal and why the deception was necessary. To additionally ensure ethically responsible research, the potentially negative task (debate) was communicated from the beginning and there was always the option to cancel (without any disadvantages).

### Conclusion

Why do people feel attracted to populist movements? In this article, we provided the first causal evidence for the control function of populist thinking and action. In addition, the novel experimental paradigm of manipulating populist attitudes critically advances research on the motivational drivers and consequences of populism as it allows for testing the causal effects of populism. Finally, our findings pave the way for novel and more specific interventions to counter a rise of populism through providing citizens with alternative sources of control, for instance, by empowering them to exert personal control in democratic decision-making.

## Supplemental Material

sj-docx-1-psp-10.1177_01461672251373106 – Supplemental material for The Control Motivation Function of Populist Attitudes: Causal Evidence that Populist Reasoning Raises Perceptions of Control Supplemental material, sj-docx-1-psp-10.1177_01461672251373106 for The Control Motivation Function of Populist Attitudes: Causal Evidence that Populist Reasoning Raises Perceptions of Control by Annedore Hoppe, Immo Fritsche and Helena Pauen in Personality and Social Psychology Bulletin

sj-docx-2-psp-10.1177_01461672251373106 – Supplemental material for The Control Motivation Function of Populist Attitudes: Causal Evidence that Populist Reasoning Raises Perceptions of Control Supplemental material, sj-docx-2-psp-10.1177_01461672251373106 for The Control Motivation Function of Populist Attitudes: Causal Evidence that Populist Reasoning Raises Perceptions of Control by Annedore Hoppe, Immo Fritsche and Helena Pauen in Personality and Social Psychology Bulletin

sj-docx-3-psp-10.1177_01461672251373106 – Supplemental material for The Control Motivation Function of Populist Attitudes: Causal Evidence that Populist Reasoning Raises Perceptions of Control Supplemental material, sj-docx-3-psp-10.1177_01461672251373106 for The Control Motivation Function of Populist Attitudes: Causal Evidence that Populist Reasoning Raises Perceptions of Control by Annedore Hoppe, Immo Fritsche and Helena Pauen in Personality and Social Psychology Bulletin

sj-docx-4-psp-10.1177_01461672251373106 – Supplemental material for The Control Motivation Function of Populist Attitudes: Causal Evidence that Populist Reasoning Raises Perceptions of Control Supplemental material, sj-docx-4-psp-10.1177_01461672251373106 for The Control Motivation Function of Populist Attitudes: Causal Evidence that Populist Reasoning Raises Perceptions of Control by Annedore Hoppe, Immo Fritsche and Helena Pauen in Personality and Social Psychology Bulletin

sj-jpg-5-psp-10.1177_01461672251373106 – Supplemental material for The Control Motivation Function of Populist Attitudes: Causal Evidence that Populist Reasoning Raises Perceptions of Control Supplemental material, sj-jpg-5-psp-10.1177_01461672251373106 for The Control Motivation Function of Populist Attitudes: Causal Evidence that Populist Reasoning Raises Perceptions of Control by Annedore Hoppe, Immo Fritsche and Helena Pauen in Personality and Social Psychology Bulletin

sj-jpg-6-psp-10.1177_01461672251373106 – Supplemental material for The Control Motivation Function of Populist Attitudes: Causal Evidence that Populist Reasoning Raises Perceptions of Control Supplemental material, sj-jpg-6-psp-10.1177_01461672251373106 for The Control Motivation Function of Populist Attitudes: Causal Evidence that Populist Reasoning Raises Perceptions of Control by Annedore Hoppe, Immo Fritsche and Helena Pauen in Personality and Social Psychology Bulletin

sj-jpg-7-psp-10.1177_01461672251373106 – Supplemental material for The Control Motivation Function of Populist Attitudes: Causal Evidence that Populist Reasoning Raises Perceptions of Control Supplemental material, sj-jpg-7-psp-10.1177_01461672251373106 for The Control Motivation Function of Populist Attitudes: Causal Evidence that Populist Reasoning Raises Perceptions of Control by Annedore Hoppe, Immo Fritsche and Helena Pauen in Personality and Social Psychology Bulletin

## Notes

1. Control (composite score): *M* = 3.59 (populism), *SD* = 0.59 versus *M* = 3.50, *SD* = 0.56, *t*(119) = −0.85, *p* = .40, *Cohen’s d* = −0.16, CI = [−0.51, 0.20]; control over the debate: *M* = 3.47, *SD* = 0.65 versus *M* = 3.35, *SD* = 0.73, *t*(119) = −0.93, *p* = .36, *Cohen’s d* = −0.17, CI = [−0.53, 0.19]; control over the retirement discourse: *M* = 3.35, *SD* = 0.85 versus *M* = 3.54, *SD* = 0.82, *t*(119) = 1.22, *p* = .224, *Cohen’s d* = 0.22, CI = [−0.14, 0.58]; control over life: *M* = 3.95, *SD* = 0.75 versus *M* = 3.75, *SD* = 0.70, *t*(119) = −1.56, *p* = .12, *Cohen’s d* = −0.28, CI = [−0.64, 0.08]; control through the ingroup: *M* = 3.67, *SD* = 0.85 versus *M* = 3.58, *SD* = 0.72, *t(*119) = −0.68, *p* = .50, *Cohen’s d* = −0.13, CI = [−0.48, 0.23].

2. Control, composite score: *M* = 4.82 (populism), *SD* = 0.67 versus *M* = 3.74, *SD* = 0.63, *t*(209) = −0.90, *p* = .37, *Cohen’s d* = −0.12, CI = [−0.39, 0.15]; control over the debate: *M* = 4.58, *SD* = 1.01 versus *M* = 3.46, *SD* = 1.01, *t*(209) = −0.84, *p* = .40, *Cohen’s d* = −0.12, CI = [−0.39, 0.16]; control over the retirement discourse: *M* = 4.27, *SD* = 1.07 versus *M* = 4.11, *SD* = 1.01, *t*(209) = −1.06, *p* = .29, *Cohen’s d* = −0.15, CI = [−0.42, 0.12]; control over life: *M* = 5.39, *SD* = 0.95 versus *M* = 5.44, *SD* = 0.81, *t*(209) = 0.40, *p* = .69, *Cohen’s d* = −0.05, CI = [−0.22, 0.33]; control through the ingroup: *M* = 4.85, *SD* = 0.94 versus *M* = 4.72, *SD* = 0.88, *t*(209) = −1.08, *p* = .28, *Cohen’s d* = −0.15, CI = [−0.42, 0.12].

3. Main effect of control salience on populism criteria: *F*(1,392) = 2.70, *p* = .101, η_p_^2^ = .01, CI = [.00, .03], interactive effect of Control Salience × Populist Attitudes on populism criteria: *F*(1,392) = 0.79, *p* = .37, η_p_^2^ = .002, CI = [.00, .02], main effect of control salience on nonpopulism criteria: *F*(1,392) = 1.28, *p* = .26, η_p_^2^ = .003, CI = [.00, .02], interactive effect of Control Salience × Populist Attitudes on nonpopulism criteria: *F*(1,392) = 0.27, *p* = .61, η_p_^2^ = .001, CI = [.00, .02].

4. Main effect of control salience: *F*(1,392) = 1.19, *p* = .277, η_p_^2^ = .00, CI = [.00, .02]; main effects of populist attitudes: *F*(1,392) = 0.11, *p* = .74, η_p_^2^ = .00, CI = [.00, .02]; interactive effect of Control Salience × Populist Attitudes: *F*(1,392) = 0.01, *p* = .94, η_p_^2^ = .00, CI = [.00, .00].

5. Main effect of control salience: *F*(1,392) = 1.76, *p* = .186, η_p_^2^ = .00, CI = [.00, .03]; main effects of populist attitudes: *F*(1,392) = 0.01, *p* = .93, η_p_^2^ = .00, CI = [.00, .01]; interactive effect of control salience x populist attitudes: *F*(1,392) = 0.44, *p* = .51, η_p_^2^ = .00, CI = [.00, .02].

6. Nonpopulism—low control versus nonpopulism—high control: *t*(391) = 1.55, *p*_Tukey_ = .41, *Cohen’s d* = .22, CI = [.06, .50]; nonpopulism—low control versus populism—high control salient: *t*(391) = −0.80, *p*_Tukey_ = .85, *Cohen’s d* = — 0.11, CI = [−.38, .16]; nonpopulism—high control versus populism—low control: *t*(391) = −1.20, *p*_Tukey_ = .63, *Cohen’s d* = .18, CI = [−.11, .46]; populism—low control versus populism—high control: *t*(391) = −1.11, *p*_Tukey_ = .68, *Cohen’s d* = −0.16, CI = [−.44, .12].

7. Nonpopulism—low control versus nonpopulism—high control: *t*(392) = 2.03, *p*_Tukey_ = .18, *Cohen’s d* = .29, CI = [.01, .57]; nonpopulism—low control versus populism—low control: *t*(392) = 0.53, *p*_Tukey_ = .95, *Cohen’s d* = .08, CI = [.21, .36]; nonpopulism—low control versus populism—high control: *t*(392) = −0.24, *p*_Tukey_ = .995, *Cohen’s d* = −.03, CI = [.31, .24]; nonpopulism—high control versus populism—low control: *t*(392) = −1.46, *p*_Tukey_ = .46, *Cohen’s d* = .21, CI = [.07, .50]; nonpopulism—high control versus populism—high control: *t*(392) = −2.29, *p*_Tukey_ = .10, *Cohen’s d* = −.32, CI = [−.60, −.05], populism—low control versus populism—high control: *t*(392) = −0.77, *p*_Tukey_ = .87, *Cohen’s d* = −.11, CI = [−.39, .17].

8. Main effect of control salience: *F*(1,391) = 0.00, *p* = .996, η_p_^2^ = .00, CI = [.00, .00], interactive effect of Control Salience × Populist Attitudes: *F*(1,391) = 0.16, *p* = .69, η_p_^2^ = .00, CI = [.00, .01].

9. Main effect of control salience, *F*(1,392) = 0.44, *p* = .51, η_p_^2^ = .00, CI = [.00, .02], interactive effect of Control Salience × Populist Attitudes: *F*(1,392) = 0.46, *p* = .50, η_p_^2^ = .00, CI = [.00, .02].

10. Nonpopulism—low control versus nonpopulism—high control: *t*(391) = 1.43, *p*_Tukey_ = .48, *Cohen’s d* = .20, CI = [.08, .49]; nonpopulism—low control versus populism—low control: *t*(391) = 1.23, *p*_Tukey_ = .61, *Cohen’s d* = .18, CI = [.11, .46]; nonpopulism—low control versus populism—high control: *t*(391) = −0.64, *p*_Tukey_ = .92, *Cohen’s d* = −.09, CI = [.36, .18]; nonpopulism—high control versus populism—low control: *t*(391) = −1.19, *p*_Tukey_ = .998, *Cohen’s d* = .03, CI = [.26, .31]; nonpopulism—high control versus populism—high control: *t*(391) = −2.09, *p*_Tukey_ = .16, *Cohen’s d* = —.29, CI = [−.57, −.01]; populism—low control versus populism—high control: *t*(391) = −1.87, *p*_Tukey_ = .24, *Cohen’s d* = −.27, CI = [−.55, .01].

11. Main effect of control salience: *F*(1,391) = 0.09, *p* = .76, η_p_^2^ = .00, CI = [.00, .01], main effect of populist attitudes: *F*(1,391) = 0.33, *p* = .56, η_p_^2^ = .00, CI = [.00, .02].

12. Main effect of control salience: *F*(1,392) = 0.01, *p* = .93, η_p_^2^ = .00, CI = [.00, .01], main effect of populist attitudes: *F*(1,392) = 0.31, *p* = .58, η_p_^2^ = .00, CI = [.00, .02].

13. ANCOVA with covariate: main effect of control salience: *F*(1,391) = 0.17, *p* = .68, η_p_^2^ = .00, CI = [.00, .01]; main effect of populist attitudes: *F*(1,391) = 0.00, *p* = .96, η_p_^2^ = .00, CI = [.00, .00]; interactive effect: *F*(1,391) = 1.43, *p* = .23, η_p_^2^ = .00, CI = [.00, .02]; ANOVA without covariate: main effect of control salience: *F*(1,392) = 0.85, *p* = .36, η_p_^2^ = .00, CI = [.00, .02]; main effect of populist attitudes: *F*(1,392) = 0.00, *p* = .996, η_p_^2^ = .00, CI = [.00, .00]; interactive effect: *F*(1,392) = 1.86, *p* = .17, η_p_^2^ = .01, CI = [.00, .03].

14. ANCOVA with covariate: main effect of control salience: *F*(1,391) = 0.75, *p* = .39, η_p_^2^ = .00, CI = [.00, .02]; main effect of populist attitudes: *F*(1,391) = 0.28, *p* = .60, η_p_^2^ = .00, CI = [.00, .02]; interactive effect: *F*(1,391) = 1.24, *p* = .27, η_p_^2^ = .00, CI = [.00, .02]; ANOVA without covariate: main effect of control salience: *F*(1,392) = 1.43, *p* = .23, η_p_^2^ = .00, CI = [.00, .02]; main effect of populist attitudes: *F*(1,392) = 0.24, *p* = .63, η_p_^2^ = .00, CI = [.00, .01]; interactive effect: *F*(1,392) = 1.57, *p* = .21, η_p_^2^ = .00, CI = [.00, .03].
