# Supplementary material for: The Control Motivation Function of Populist Attitudes: Causal Evidence that Populist Reasoning Raises Perceptions of Control
Source: Pers Soc Psychol Bull. 2025 Oct 31;52(10):3512–27. doi: 10.1177/01461672251373106 (PMC13507497; doi:10.1177/01461672251373106)
Supplement: Supplementary file 1 [file sj-docx-1-psp-10.1177_01461672251373106.docx]

| Table S1 | | | | | | |
| --- | --- | --- | --- | --- | --- | --- |
| **Preregistration deviations** | | | | | | |
| # | Details | | Original wording | Deviation description | To what extent is this a deviation from the preregistered plan? | Judgment of impact |
| 1 | Type | Analyses | *Independent samples t-tests with 2 groups will be conducted* | *We conducted Ancovas and included feeling comfortable with the way of reasoning as a covariate.* | Minor | *People differed in the extend they comfortable with the manipulation. We did not expect that (initially, we planned t-tests without covariate).* |
|  | Reason | New knowledge |  |  |  |  |
|  | Timing | After results known |  |  |  |  |
| 2 | Type | Analyses | *observations will be*  *excluded if*  *- an error in the data entry / measurement occurred*  *- a participant left more than 10 % of the items measuring the DV unanswered*  *- a participant takes less than 1.5 minutes to complete the reasoning task (despite being asked to take at least 3 minutes).* | *We did not exclude those participants.* | Minor | *No impact, results are similar when those participants are excluded. In detail:*   - *main effect on overall control after excluding (p = .004 vs. p = .024 if not excluded)* - *effect on control over the debate after excluding (p = .017 vs. p = .032 if not excluded)* - *effect on control over the retirement discourse (p = .60 vs. p = .93 if not excluded)* - *effect on control over life after excluding (p = .010 vs p = .002 if not excluded)* - *effect on control through the ingroup after excluding (p = .072 vs p = .080 if not excluded)* |
|  | Reason | Miscommunication |  |  |  |  |
|  | Timing | After data access |  |  |  |  |
| 3 | Type | Analyses | *Effects of populist vs. non-populist reasoning were expected on all 4 subscales* | *We did not find effects on the subscale “control over the societal retirement discourse” and only marginally significant effects on the subscale “control through the ingroup”* | Minor | *Missing effects might be due to the low intensity of the (online) manipulation; this issue is taken up in Study 2.* |
|  | Reason | New knowledge |  |  |  |  |
|  | Timing | Select One |  |  |  |  |

*Note.* Wilroth, E. C., & Atherton, O. E. (in press). Best laid plans: A guide to reporting preregistration deviations. *Advances in Methods and Practices in Psychological Science.* Retrieved from <https://osf.io/preprints/psyarxiv/dwx69>
