# Supplementary material for: The Control Motivation Function of Populist Attitudes: Causal Evidence that Populist Reasoning Raises Perceptions of Control
Source: Pers Soc Psychol Bull. 2025 Oct 31;52(10):3512–27. doi: 10.1177/01461672251373106 (PMC13507497; doi:10.1177/01461672251373106)
Supplement: Supplementary file 2 [file sj-docx-2-psp-10.1177_01461672251373106.docx]

| Table S2 | | | | | | |
| --- | --- | --- | --- | --- | --- | --- |
| **Preregistration deviations** | | | | | | |
| # | Details | | Original wording | Deviation description | To what extent is this a deviation from the preregistered plan? | Judgment of impact |
| 1 | Type | Analyses | *The effects [of populist attitudes] should be seen mainly in terms of the perception of influence over one's own life, less in terms of more specific measures of personal control.* | *We found effects on subscales representing more immediate control measures, but no significant effects on control over life.* | Minor | *Probably, the immersive lab manipulation (vs. online in Study 1) led participants to focus more strongly on their immediate goal of representing their own position in the announced group discussion and on its specific content (compared to the online setting in Study 1), thus increasing effects on more immediate control measures and reducing effects on more distal measures.* |
|  | Reason | New knowledge |  |  |  |  |
|  | Timing | After results known |  |  |  |  |
| 2 | Type | Analyses | *Main effects will be analyzed using ANOVAS.* | *We conducted ANCOVAS and included feeling comfortable with the way of reasoning as a covariate.* | Minor | *People differed in the extend to which they felt comfortable with the manipulation. We did not expect that (initially, we planned t-tests without any covariate).* |
|  | Reason | New knowledge |  |  |  |  |
|  | Timing | After data access |  |  |  |  |

*Note.* Wilroth, E. C., & Atherton, O. E. (in press). Best laid plans: A guide to reporting preregistration deviations. *Advances in Methods and Practices in Psychological Science.* Retrieved from <https://osf.io/preprints/psyarxiv/dwx69>
