# Supplementary material for: The Control Motivation Function of Populist Attitudes: Causal Evidence that Populist Reasoning Raises Perceptions of Control
Source: Pers Soc Psychol Bull. 2025 Oct 31;52(10):3512–27. doi: 10.1177/01461672251373106 (PMC13507497; doi:10.1177/01461672251373106)
Supplement: Supplementary file 3 [file sj-docx-3-psp-10.1177_01461672251373106.docx]

| Table S3 | | | | | | |
| --- | --- | --- | --- | --- | --- | --- |
| **Preregistration deviations** | | | | | | |
| # | Details | | Original wording | Deviation description | To what extent is this a deviation from the preregistered plan? | Judgment of impact |
| 1 | Type | Interpretation | *We expect that control perceptions are enhanced after an induced threat to personal control when participants can restore their control (vs. not) through populist actions.* | *Effect of populist (vs. non-populist) attitudes is eliminated under salience of low control, in particular with regard to the subscale control over life.* | Major | *Under salience of low control, people might be motivated to bias their control perceptions upwards. Acting in a populist way might buffer this reaction. Similar to our hypothesis, populist reasoning may serve to enhance personal control, but the pattern of results is different. This should be examined in future studies.* |
|  | Reason | New knowledge |  |  |  |  |
|  | Timing | After results known |  |  |  |  |

*Note.* Wilroth, E. C., & Atherton, O. E. (in press). Best laid plans: A guide to reporting preregistration deviations. *Advances in Methods and Practices in Psychological Science.* Retrieved from <https://osf.io/preprints/psyarxiv/dwx69>
