# Supplementary material for: The Control Motivation Function of Populist Attitudes: Causal Evidence that Populist Reasoning Raises Perceptions of Control
Source: Pers Soc Psychol Bull. 2025 Oct 31;52(10):3512–27. doi: 10.1177/01461672251373106 (PMC13507497; doi:10.1177/01461672251373106)
Supplement: Supplementary file 4 [file sj-docx-4-psp-10.1177_01461672251373106.docx]

| **Table S4** | | | | | | | |
| --- | --- | --- | --- | --- | --- | --- | --- |
| Correlation Table (Study 1) | | | | | | | |
|  | 1 - Control (Composite Score) | 2 – Control over the debate | 3 – Control over the societal discourse | 4 – Control over life | 5 – Control through the ingroup | 6 – Populist attitudes | 7 – Feeling comfortable |
| 1 | — |  |  |  |  |  |  |
| 2 | 0.908*** | — |  |  |  |  |  |
| 3 | 0.700*** | 0.541*** | — |  |  |  |  |
| 4 | 0.699*** | 0.452*** | 0.429*** | — |  |  |  |
| 5 | 0.664*** | 0.457*** | 0.324*** | 0.385*** | — |  |  |
| 6 | 0.097 | 0.003 | 0.182* | 0.056 | 0.189* | — |  |
| 7 | 0.378*** | 0.331*** | 0.364*** | 0.181* | 0.288** | 0.335*** | — |
| Note. * p < .05, ** p < .01, *** p < .001 | | | | | | | |

| **Table S5** | | | | | | | |
| --- | --- | --- | --- | --- | --- | --- | --- |
| Correlation Table (Study 2) | | | | | | | |
|  | 1 - Control (Composite Score) | 2 – Control over the debate | 3 – Control over the societal discourse | 4 – Control over life | 5 – Control through the ingroup | 6 – Populist attitudes | 7 – Feeling comfortable |
| 1 | — |  |  |  |  |  |  |
| 2 | 0.775*** | — |  |  |  |  |  |
| 3 | 0.656*** | 0.475*** | — |  |  |  |  |
| 4 | 0.677*** | 0.290*** | 0.230*** | — |  |  |  |
| 5 | 0.589*** | 0.228*** | 0.158* | 0.275*** | — |  |  |
| 6 | 0.015 | -0.051 | -0.016 | 0.068 | 0.049 | — |  |
| 7 | 0.311*** | 0.326*** | 0.239*** | 0.114 | 0.151* | 0.195** | — |
| Note. * p < .05, ** p < .01, *** p < .001 | | | | | | | |

| **Table S6** | | | | | | | |
| --- | --- | --- | --- | --- | --- | --- | --- |
| Correlation Table (Study 3) | | | | | | | |
|  | 1 - Control (Composite Score) | 2 – Control over the debate | 3 – Control over the societal discourse | 4 – Control over life | 5 – Control through the ingroup | 6 – Populist attitudes | 7 – Feeling comfortable |
| 1 | — |  |  |  |  |  |  |
| 2 | 0.769*** | — |  |  |  |  |  |
| 3 | 0.723*** | 0.504*** | — |  |  |  |  |
| 4 | 0.700*** | 0.338*** | 0.288*** | — |  |  |  |
| 5 | 0.716*** | 0.367*** | 0.389*** | 0.349*** | — |  |  |
| 6 | 0.413*** | 0.308*** | 0.294*** | 0.190*** | 0.422*** | — |  |
| 7 | 0.459*** | 0.494*** | 0.414*** | 0.164** | 0.274*** | 0.383*** | — |
| Note. * p < .05, ** p < .01, *** p < .001. Additionally controlling for populist attitudes (Schulz scale, in addition to feeling comfortable) does not change the interaction effect of threat x populist attitudes on control perceptions (composite score), *F*(1,390) = 3.61, *p* = .058, *η_p_^2^* = .01, or the main effect of populist attitudes on control through the slogans, *F*(1,390) = 5.94, *p* = .015, *η_p_^2^* = .02. | | | | | | | |
